# Supplementary figures and images for: Influence of Neighborhood Size and Cross-Correlation Peak-Fitting Method on Location Accuracy
Source: Sensors (Basel). 2020 Nov 18;20(22):6596. doi: 10.3390/s20226596 (PMC7698887; doi:10.3390/s20226596)

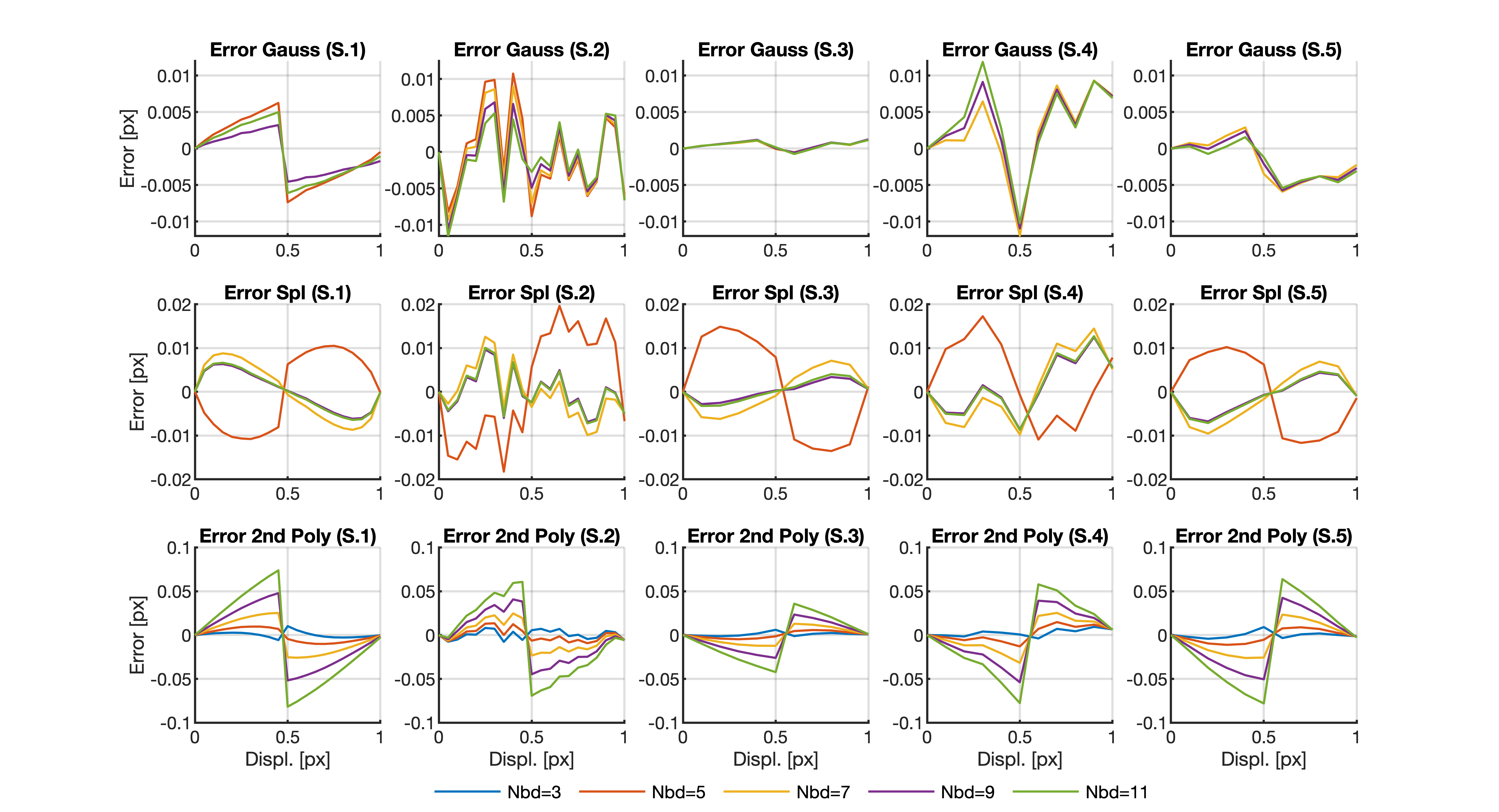

Supplement: Supplementary file 1 [file sensors-20-06596-s001.zip › Fig3.png]

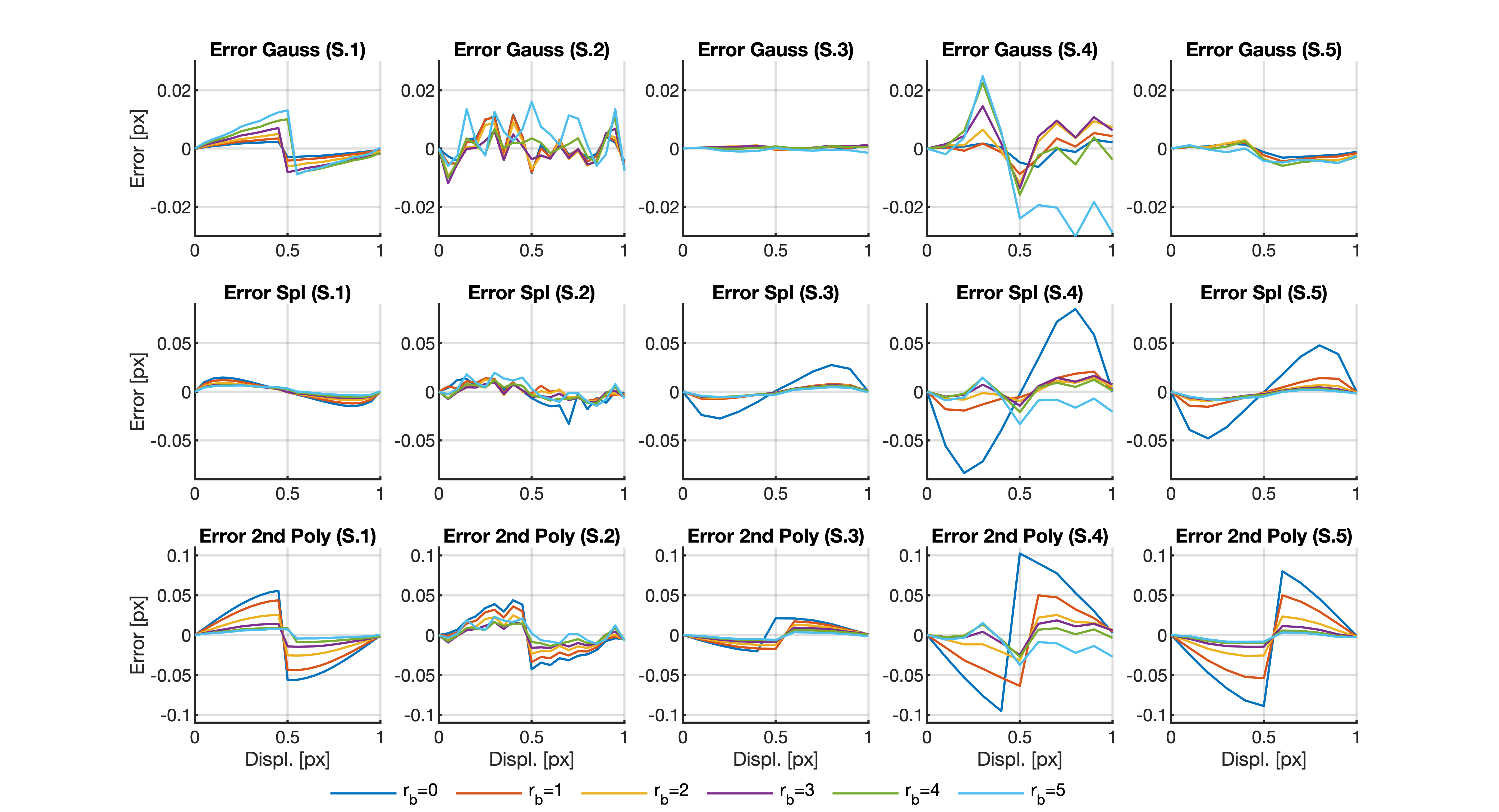

Supplement: Supplementary file 1 [file sensors-20-06596-s001.zip › Fig4.png]

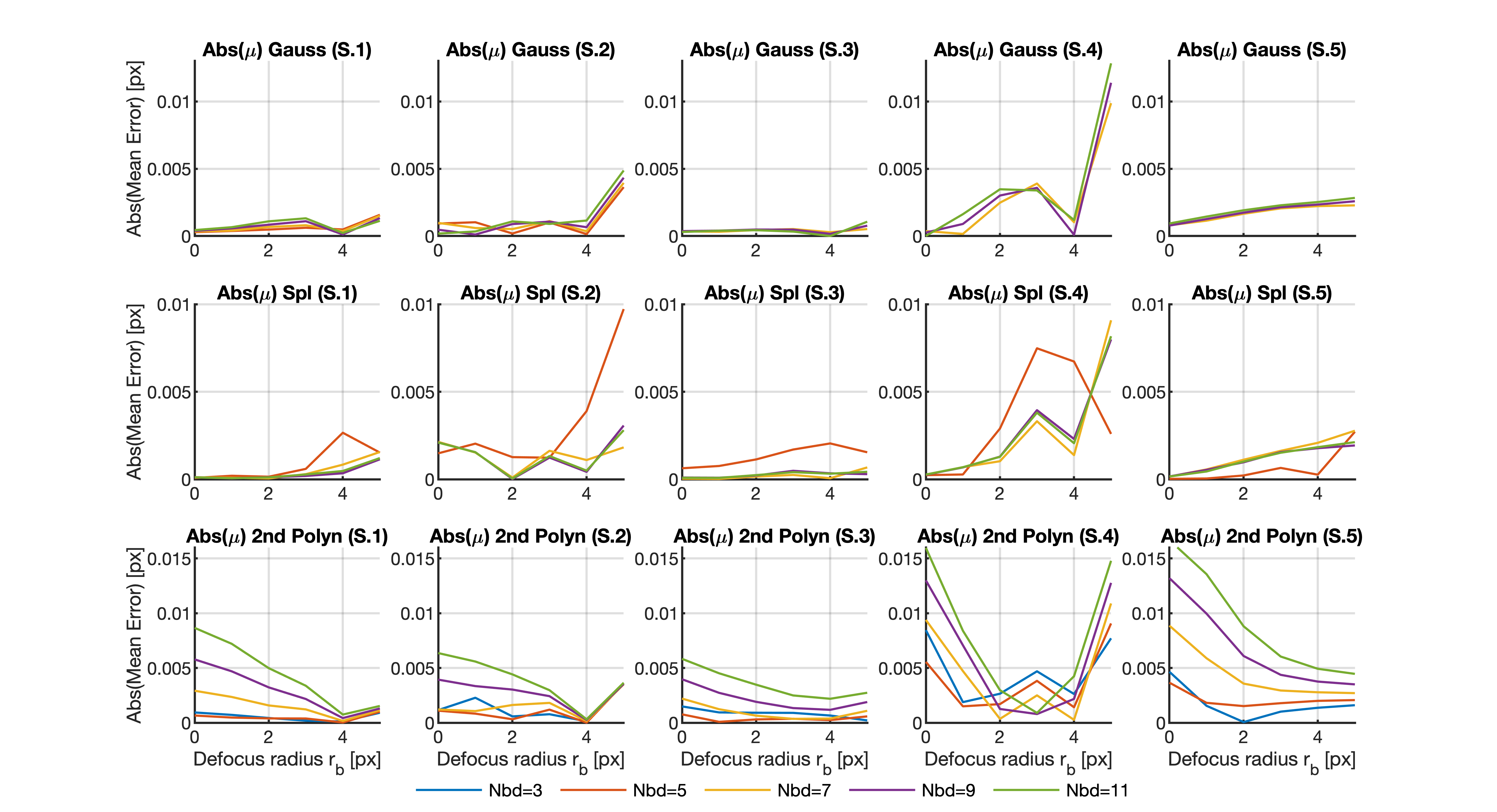

Supplement: Supplementary file 1 [file sensors-20-06596-s001.zip › Fig5.png]

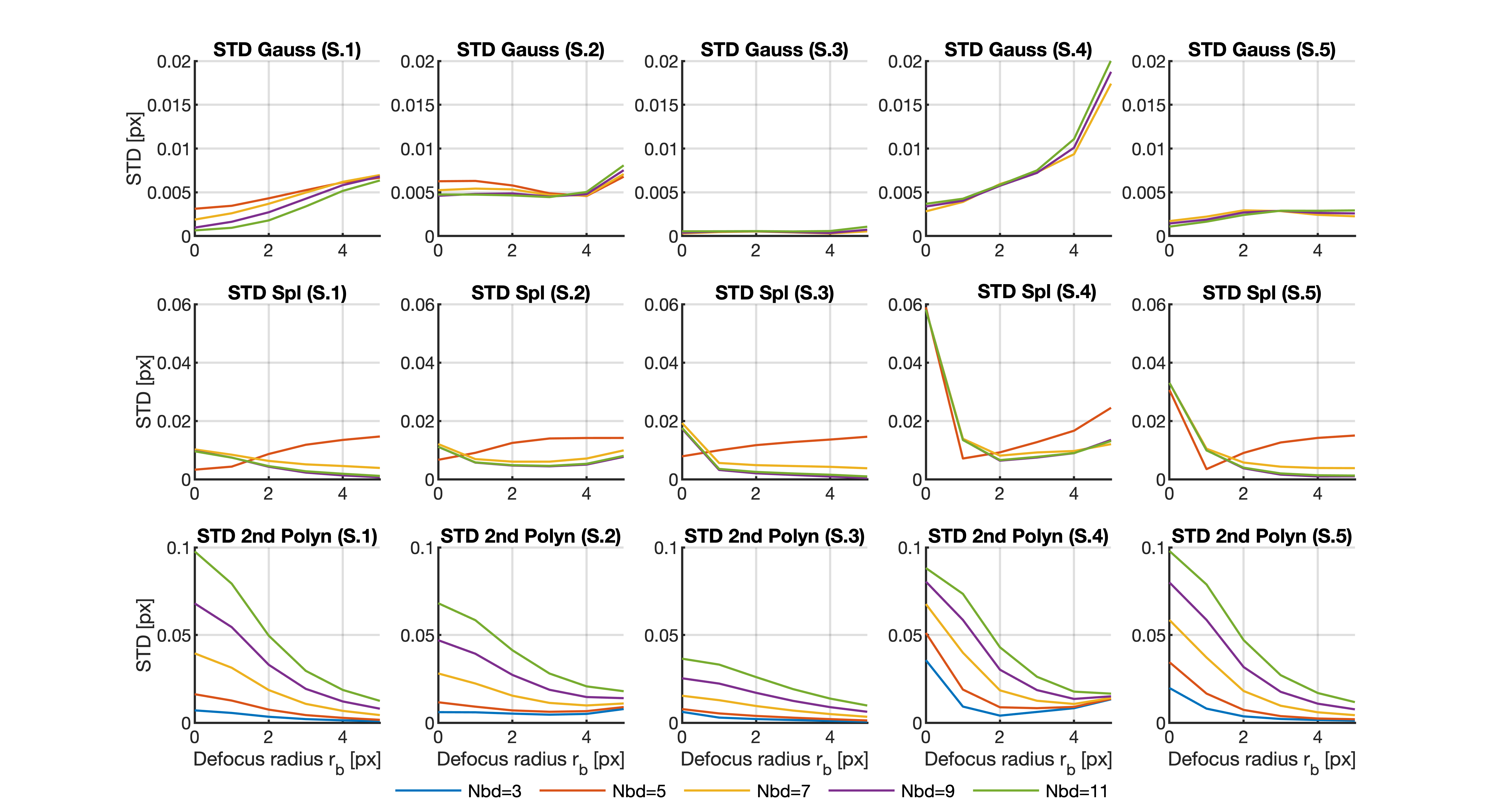

Supplement: Supplementary file 1 [file sensors-20-06596-s001.zip › Fig6.png]

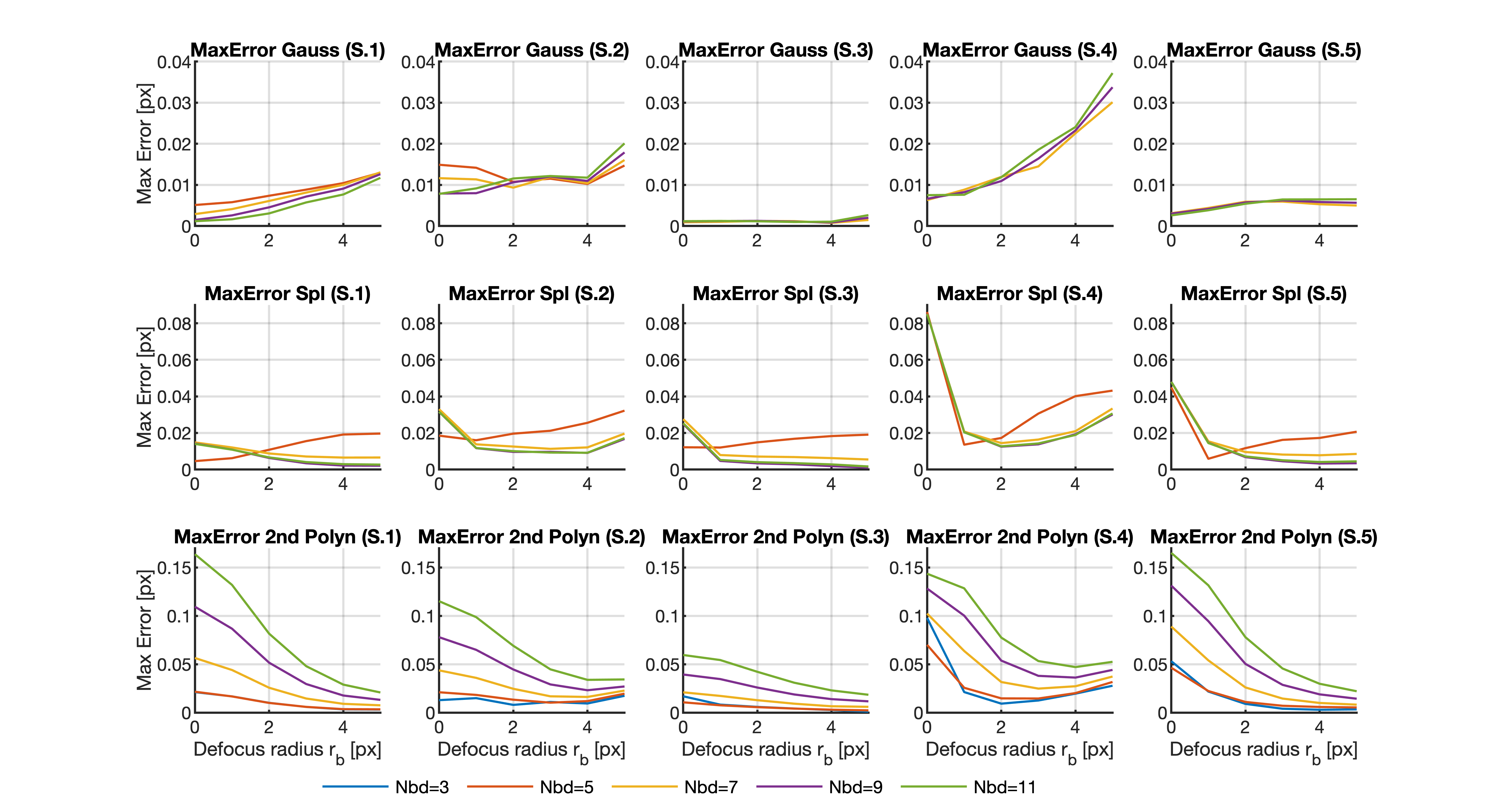

Supplement: Supplementary file 1 [file sensors-20-06596-s001.zip › Fig7.png]

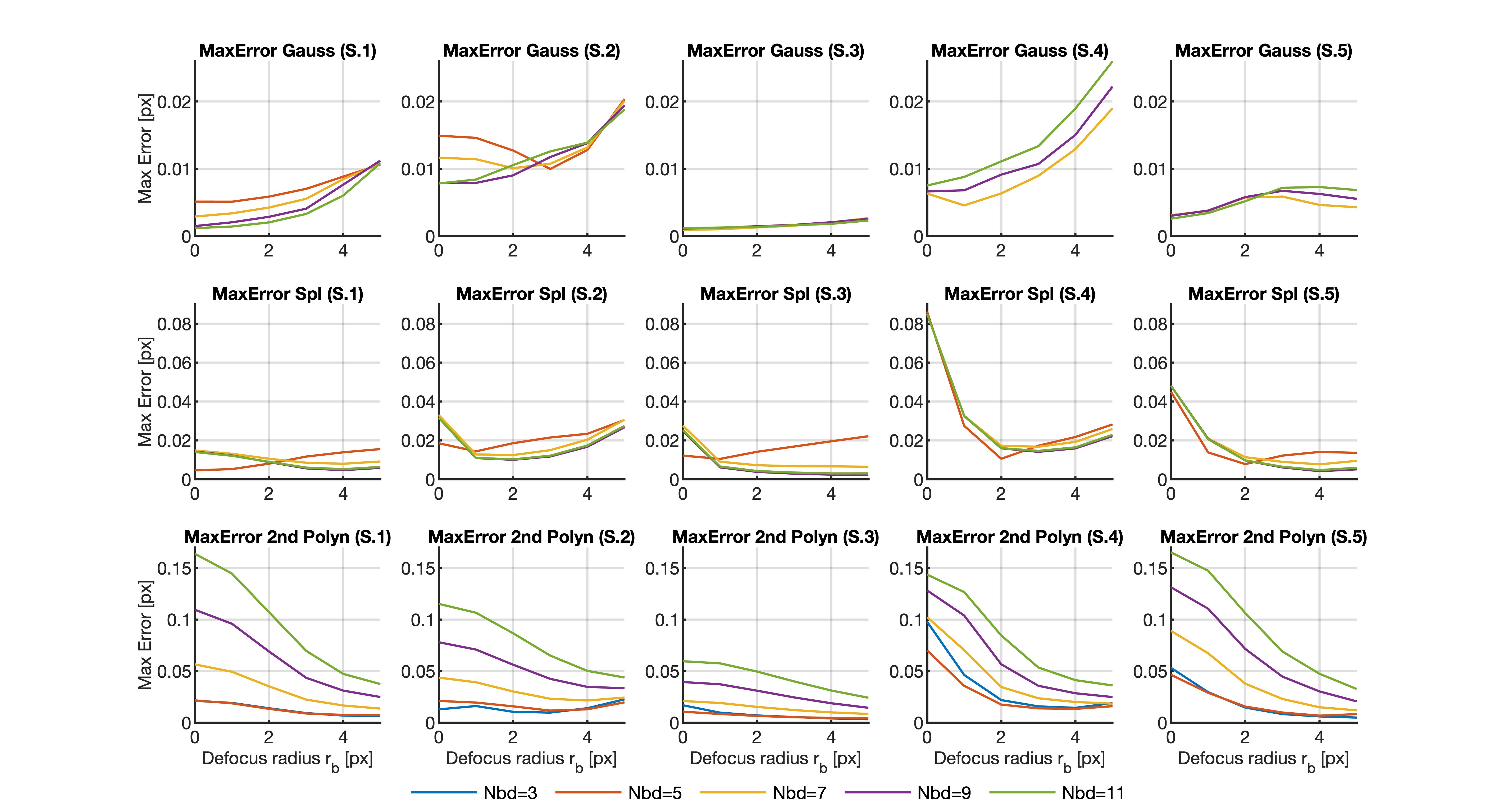

Supplement: Supplementary file 1 [file sensors-20-06596-s001.zip › Fig9.png]
